# Genetic and Neurobiological Analyses of the Noradrenergic-like System in Vulnerability to Sugar Overconsumption Using a *Drosophila* Model

**DOI:** 10.1038/s41598-017-17760-w

**Published:** 2017-12-15

**Authors:** Audrey Branch, Yiwen Zhang, Ping Shen

**Affiliations:** Department of Cellular Biology and Biomedical and Health Sciences Institute, University of Georgia, 500 D. W. Brooks Drive, Athens, GA 30602 USA

## Abstract

Regular overconsumption of sugar is associated with obesity and type-2 diabetes, but how genetic factors contribute to variable sugar preferences and intake levels remains mostly unclear. Here we provide evidence for the usefulness of a *Drosophila* larva model to investigate genetic influence on vulnerability to sugar overconsumption. Using genetic and RNA interference approaches, we show that the activity of the *Oamb* gene, which encodes a receptor for octopamine (OA, the invertebrate homologue of norepinephrine), plays a major role in controlled sugar consumption. Furthermore, Oamb appears to suppress sugar food intake in fed larvae in an acute manner, and neurons expressing this Oamb receptor do not overlap with neurons expressing Octβ3R, another OA receptor previously implicated in hunger-driven exuberant sugar intake. Together, these results suggest that two separate sub-circuits, defined by Oamb and Octβ3R respectively, co-regulate sugar consumption according to changes in energy needs. We propose that the noradrenergic-like system defines an ancient regulatory mechanism for prevention of sugar overload.

## Introduction

Sugar is a vital energy source that is highly rewarding. A carbohydrate-rich meal triggers a rapid insulin release that restores blood or hemolymph sugar to the baseline level in both mammals and invertebrates^1–3^. However, the regulatory capacity of the insulin system is limited. Long term sugar overconsumption, frequently caused by eating disorders such as binge eating in humans, will likely leads to diabetic disorders^4^. At present, our understanding of genetic and neural mechanisms underlying sugar eating disorders remains limited, partly because of the complexity of the nervous system of traditional animal models.

*Drosophila* larvae are surrounded by readily accessible sugar-rich food most of their lives. These animals appear to regulate their sugar intake and metabolism through two conserved signaling systems. First, our previous study has shown that targeted lesioning of a small subset of norepinephrine-like octopamine (OA) neurons from the larval hindbrain-like subesophageal ganglia (SOG) led to increased feeding of glucose-containing liquid food under well-nourished conditions^5^. In addition, an insulin-mediated regulatory mechanism has been identified that is essential for suppressing the surge of blood sugar level^6^. These findings have prompted us to propose that *Drosophila* larvae may offer a useful model to investigate genetic influence on the vulnerability to sugar overconsumption.

In this work, we show that the *Oamb* gene, which encodes an α-adrenergic-like receptor for OA, defines a major genetic pathway for preventing sugar overconsumption in well-nourished fly larvae. We also provide evidence that controlled intake of sugar food by larvae in adaptation to energy needs requires coordinated regulation by two distinct OA receptors, each defining a separate neural circuit. Based on these findings, we propose that the noradrenergic-like system defines an ancient regulatory mechanism for prevention of sugar overload.

## Results

### Conditional knockdown of an OA receptor activity led to sugar overconsumption

**Figure 1:**
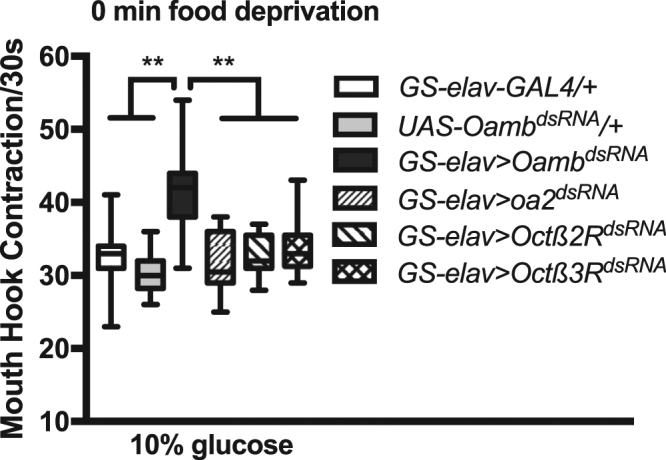
Conditional knockdown of *Oamb* activity in the nervous system leads to increased feeding of sugar food in well-nourished larvae. Glucose feeding rate of fed larvae was increased after conditional knockdown of receptor Oamb in the nervous system. For this and other figures, feeding activities were scored under blind conditions. Kruskal-Wallis test was used followed by Dunn’s multiple comparisons test: **P < 0.001, n = 10–35.

The fly genome encodes an α-adrenergic-like receptor Oamb (or Oa1) and three β-adrenergic-like receptors, Octβ1R (or Oa2), Octβ2R, and Octβ3R^7,8^. Given that lesioning of OA neurons in the SOG led to sugar food overconsumption in fed larvae, we decided to probe the potential regulatory roles of OA receptors in controlled sugar intake by conditionally knocking down the activity of each of the four receptors. This was achieved by expressing the double-stranded RNA of each receptor using a mifepristone-inducible pan-neural *GS-elav-GAL4* in fed larvae. We found that functional knockdown of Oamb, but not other subtypes, led to a significant increase in larval feeding response to 10% glucose liquid food (Fig. 1), suggesting that the normal *Oamb* receptor expression in the nervous system is acutely required to prevent sugar overconsumption in fed larvae.

### Genetic analysis of regulation of sugar consumption by Oamb

**Figure 2:**
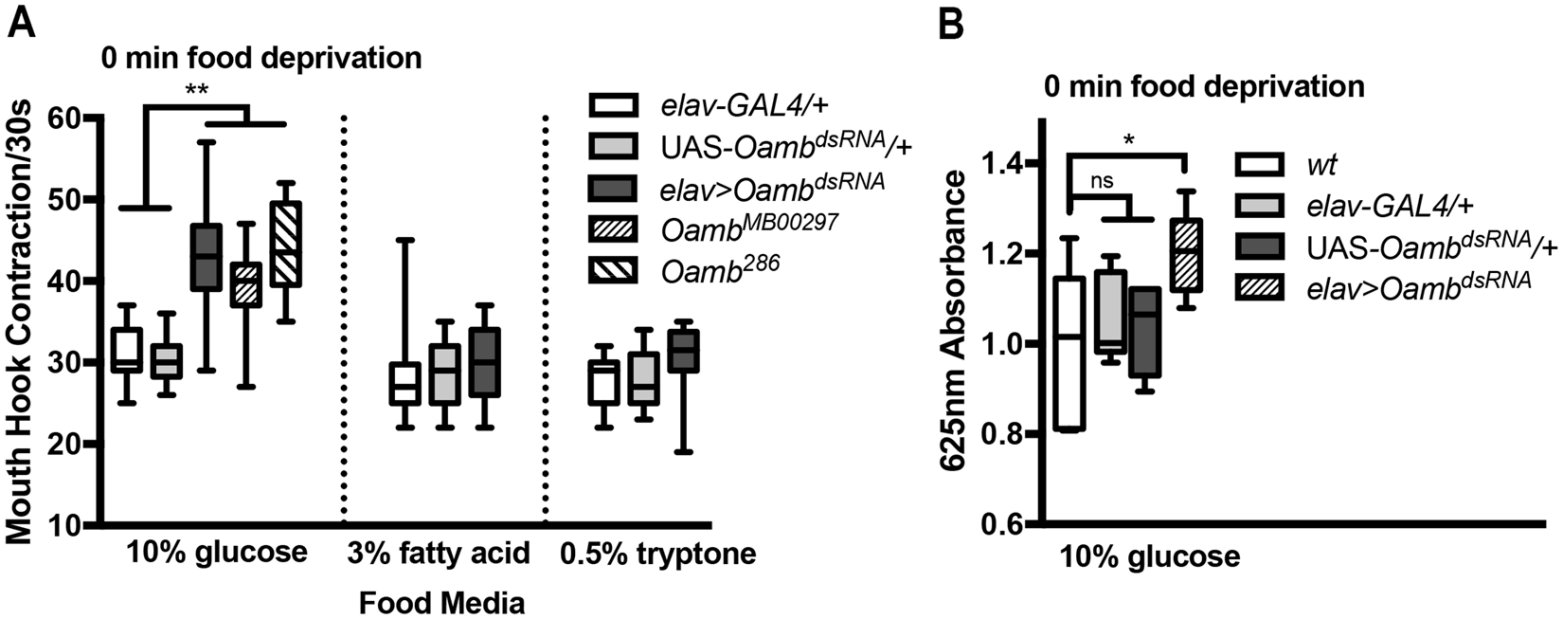
Genetic analysis of *Oamb* activity related to sugar feeding in well-nourished larvae. (**A**) *Oamb* ^286^ and *Oamb* ^*MB00297*^ (a null and an insertion allele, respectively) showed increased feeding response to the glucose medium. Pan-neural expression of the double stranded RNA (dsRNA) of *Oamb* also led to a significant increase in the glucose feeding response. Glucose assay: one-way ANOVA was used followed by Tukey’s multiple comparisons test: F(4,92) = 29.68, **P < 0.0001, n = 12–26. The feeding responses of the experimental and control larvae to oleic acid or tryptone media were at similar levels. Kruskal-Wallis test was used followed by Dunn’s multiple comparisons test, n = 15–29. (**B**) For the glucose food ingestion assay, one-way ANOVA was used followed by Dunnett’s multiple comparisons: F(3,20) = 3.427; *P = 0.0207, n = 6 batches, 30 larvae per batch.

We postulate that genetic factors including those related to the Oamb pathway may have major influences on sugar consumption, and that fly larva could be useful for investigating underlying genetic mechanisms. To test this hypothesis, we first examined how genetic manipulation of Oamb receptor expression might affect larval feeding response to sugar food. We found that in the presence of the glucose medium, both an *Oamb* insertion mutant and an Oamb null mutant showed significantly increased feeding responses under fed conditions, phenocopying the *GS-elav-GAL4/*UAS*-Oamb* ^*dsRNA*^ fed larvae (Fig. 2). In addition, *elav-GAL4/*UAS*-Oamb* ^*dsRNA*^ fed larvae, which constitutively express the *Oamb* dsRNA in the nervous system, also showed a similar increase in the feeding rate. Together, these findings suggest that genetic manipulations that result in a reduction in the Oamb pathway can have a major effect on the level of sugar consumption.

### Selective regulation of sugar/carbohydrate consumption by Oamb

These findings raised the question of whether Oamb-deficient fed larvae display excessive feeding activity in the presence of other types of palatable food. To examine this, we also tested the feeding responses of *elav-GAL4/*UAS*-Oamb* ^*dsRNA*^ fed larvae to liquid media containing 0.5% tryptone or 3% oleic acid^9,10^. We found that Oamb-deficient larvae showed a normal baseline level of feeding response to the protein- or fatty acid-rich media (Fig. 2A). Furthermore, we also directly measured the food ingestion of *elav-GAL4/*UAS*-Oamb* ^*dsRNA*^ fed larvae and controls. Again, sugar food consumption is positively correlated with mouth hook contraction rate (see Fig. 2B). Therefore, these results suggest that the Oamb receptor defines a feeding circuit that selectively prevents overconsumption of food enriched in carbohydrate but not protein or fat under well-nourished conditions.

### Functional mapping of the neural Oamb activity

**Figure 3:**
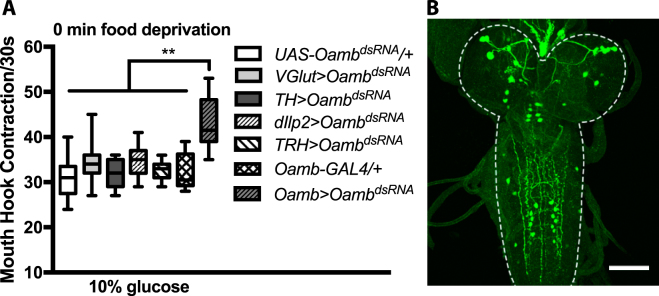
Functional knockdown of Oamb receptor activity in various subsets of neurons using different GAL4 drivers. (**A**) *Oamb-GAL4* driven Oamb knockdown mimicked pan-neural Oamb knockdown. Kruskal-Wallis test was used followed by Dunn’s multiple comparisons test: F(4,84) = 3.933, **P < 0.0001, n = 12–29. (**B**) Immunofluorescence of GFP expressed in 1.6-*Oamb-GAL4* neurons (also see Supplementary Fig. S1). The CNS tissue is outlined by white dotted line. Scale bar = 50um.

As a first step towards characterization of the underlying circuit mechanism, we first functionally knocked down *Oamb* activity in genetically defined subsets of neurons previously implicated in the control of feeding behavior under fed conditions^6,11,12^. However, expression of *Oamb* dsRNA in neurons that produce serotonin, dopamine, vesicular glutamate transporter, and insulin-like peptides failed to yield any significant increases in the glucose food response of fed larvae (Fig. 3A). Subsequently, we constructed a new *GAL4* driver (*1*.*6-Oamb-GAL4*) using a 1.6-kb promoter fragment from the Oamb gene. We found that *1*.*6-Oamb-GAL4/*UAS*-Oamb* ^*dsRNA*^ fed larvae showed a significant increase in the feeding response, similar to that of *elav-GAL4/*UAS*-Oamb* ^*dsRNA*^ fed larvae (Fig. 3A). Using a nuclear GFP reporter, we found that this line predominantly labeled a limited number of neurons in the brain lobes as well as the subesophageal and ventral ganglia (Fig. 3B).

### Functional mapping of the neural Octβ3R activity

**Figure 4:**
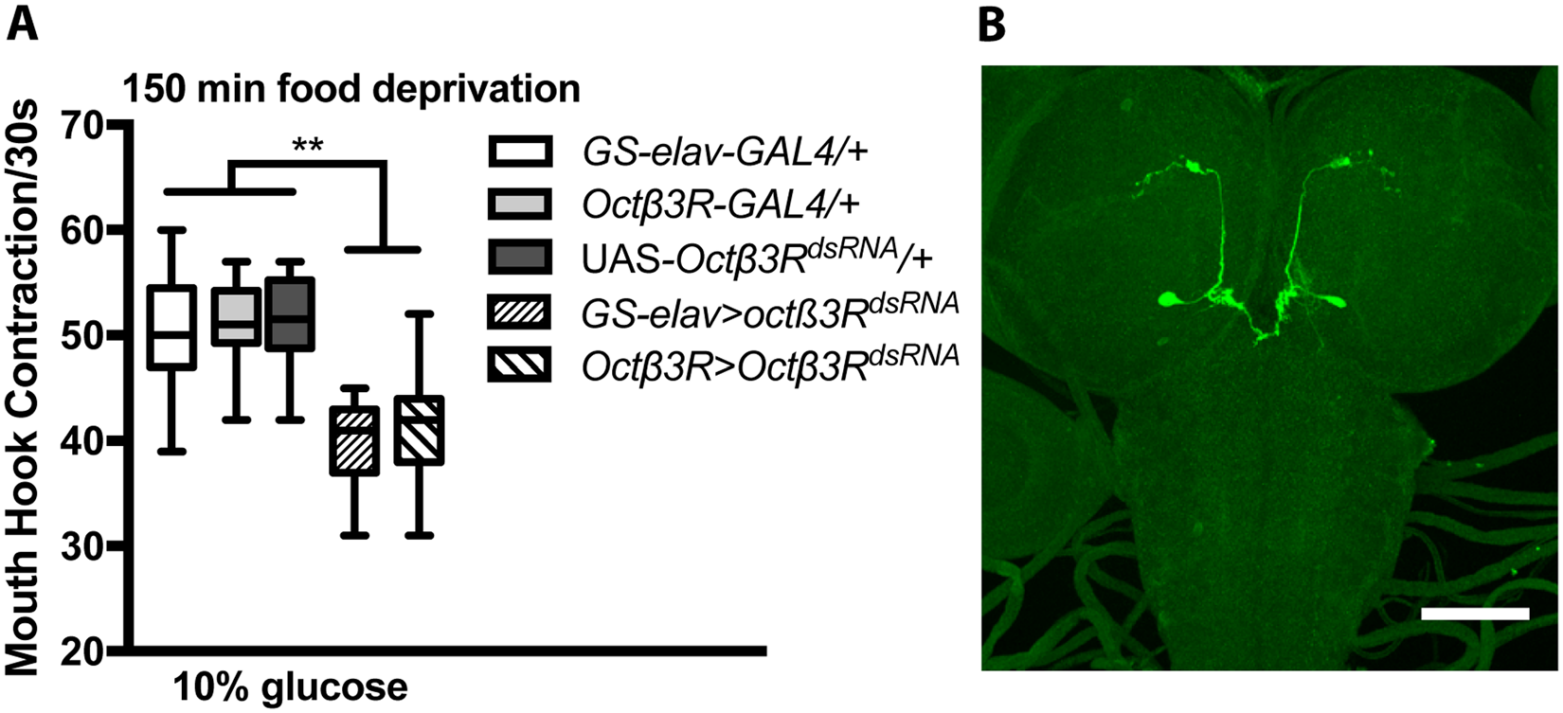
Conditional knockdown of Octβ3 R receptor activity suppressed hunger-driven increases in sugar consumption. (**A**) The rate of glucose feeding in fasted larvae was suppressed after conditional pan-neuronal knockdown of receptor Octβ3 R. Functional knockdown of Octβ3 R in 1.8-*Octβ3R-GAL4* neurons also attenuated hunger-drive feeding in fasted larvae. Kruskal-Wallis test was used followed by Dunn’s multiple comparisons test. **P < 0.01, n = 10–25. (**B**) Immunofluorescence of GFP in 1.8-*Octβ3R-GAL4* neurons (also see Supplementary Fig. S1). Scale bar = 50um.

Our previous work showed that conditional knockdown of Octβ3R, a β-adrenergic-like OA receptor, in the larval nervous system attenuated hunger-driven feeding response to sugar food^5^. To evaluate the functional relationship between the Oamb and Octβ3R circuits, we constructed a *1*.*8-Octβ3R-GAL4* driver using a 1.8-kb *Octβ3R* promoter fragment. We found that *1*.*8-Octβ3R-GAL4/*UAS*-Octβ3R* ^*dsRNA*^ larvae failed to show hunger-driven feeding of sugar food in food-deprived conditions (Fig. 4A). Furthermore, this *1*.*8-Octβ3R-GAL4* directed the GFP reporter expression in two central neurons in the tritocerebrum of larvae that do not overlap with *1*.*6-Oamb-GAL4* neurons (Fig. 4B). Together, our findings suggest that two separate OA subprograms, mediated by distinct subsets of central neurons, underlie the opposite regulatory effects of OA on sugar consumption under different motivation states (satiation and hunger).

## Discussion

We have shown that two of the four OA receptors encoded by the *Drosophila* genome mediate the dual role of the OA system in modulation of feeding of readily available sugar food under different motivational states. An α-adrenergic-like receptor Oamb is acutely required for prevention of sugar overconsumption in fed larvae, while a β-adrenergic-like receptor Octβ3R is required for hunger-driven responses to the sugar food. Our findings suggest that the adrenergic-like system of invertebrate animals is a crucial regulator that links the motivational state to the adaptive consumption of sugar, a vital energy source.

### The impact of genetic deficiencies in the *Oamb* gene on sugar consumption

Sugar food preference is known to vary among individuals, and our understanding of how genetic factors contribute to such variations remain limited^13–15^. We have shown that functional deficiency of the *Oamb* gene caused significant increases in the sugar food consumption in fed larvae. These results raise the possibility that mutations in an array of genes involved in the OA/Oamb pathway may also have similar effects on sugar food consumption. Therefore, our findings suggest that the fly larva may be a useful platform for investigating the contributions of genetic factors to variations in sugar consumption among individual animals. It would also be interesting to test whether genetic variations that affect the function of norepinephrine system may underlie the genetic predisposition to crave for sugar-rich food in mammals.

### The functional relationship between Oamb and Octβ3R sub-circuits

**Figure 5:**
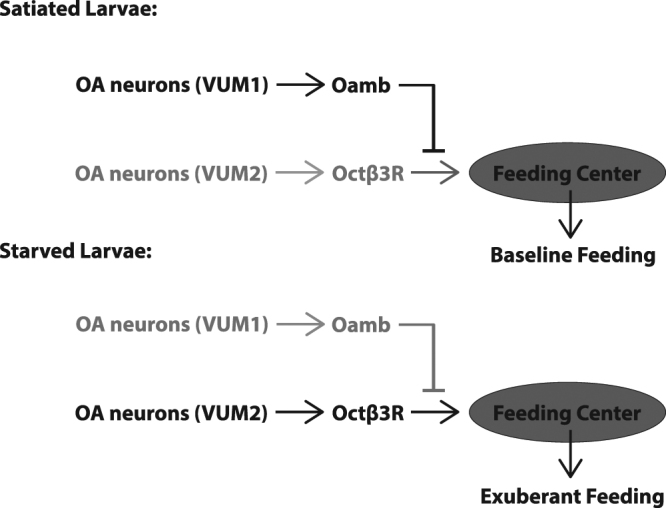
A schematic presentation of a working model for the roles of the Oamb and Octβ3 R sub-circuits in regulation of larval feeding activities under different motivational states. Active neural circuits are in black, and inactive circuits are in light grey.

Our previous study provided evidence for a potential interaction between the OA/Oamb- and OA/Octβ3R-mediated sub-circuits in modulation of sugar consumption by fly larvae^5^. It has shown that two separate subsets of OA neurons (named VUM1 and VUM2, respectively) in the hindbrain-like region are required for the control of sugar food ingestion. Targeted lesioning of VUM1 resulted in sugar overconsumption in fed larvae, while targeted lesioning of VUM2 attenuated Octβ3R-dependent feeding of sugar food in hungry larvae. Further, targeted lesioning of VUM2 also attenuated Octβ3R-dependent feeding response to sugar food. However, how VUM1 and VUM2 neurons functionally interact with each other remains unclear. In this work, our evidence supports the notion that VUM1 neurons are acutely active in fed larvae but silenced under prolonged food deprivation (Fig. 5). In fed larvae, VUM1 may indirectly suppress a VUM2-dependent sub-circuit through its signaling to Oamb neurons. It is possible that the VUM1/Oamb neuronal pathway may exert the inhibitory effect on the VUM2/Octβ3R neuronal pathway at the level of the Octβ3R neurons or their downstream targets. Further experiments will be needed to determine how the OA/Oamb and OA/Octβ3R sub-circuits interact to co-regulate sugar consumption under different motivational states.

### Control mechanisms for carbohydrates intake in flies and mammals

Carbohydrates are vital energy sources to animals across evolution. Despite considerable evolutionary divergence, the control mechanisms for carbohydrate intake in insects and mammals may share similar molecular and neural mechanisms. For example, OA neurons from the hindbrain-like SOG region are known to be associated with sugar sensation in insects. Treatment of OA promotes honey bee’s feeding response toward sucrose^16^, and is able to increase the reward value of food resources^17^. It has also been reported that OA is necessary and can even replace sugar stimuli in forming appetitive olfactory memories in Drosophila^18,19^. Similarly, a group of norepinephrine (the vertebrate counterpart of OA) neurons in the brainstem of rats are responsive to glucose level^20–22^ required for regulating carbohydrates-specific food ingestion^23^.

It is proposed that precise control of feeding is achieved through different affinities between agonists and different receptors, and the relative activity level of α1 and α2 receptor neurons determines the feeding consequences^24^. In rats, antagonistic effects of altering food intake are mediated through different downstream receptor neurons located in the paraventricular nucleus of hypothalamus^24,25^. NE signaling promotes feeding through α1 receptors^26,27^, while its activation of α2 receptors inhibits food intake^28,29^. In *Drosophila* larvae, we have also identified two separate OA circuits exerting opposite effects in regulating feeding. Similar to mammalian models, two different downstream receptors are found exhibiting antagonistic effects. Both *1*.*6-Oamb-GAL4* and 1.8-*Octβ3R-GAL4* neurons are present in a larval brain region anterior to the OA neurons. It would be interesting to determine whether this region represents a functional equivalence of the mammalian hypothalamus. Furthermore, satiation status in rats affects an animal’s feeding decisions by altering both NE release adrenoceptor levels^30,31^. We postulate that the OA system is also subject to modulation by endocrine hormones and nutrients levels, and it may define a key control site in the central nervous system where multi-sensory integration and feeding regulation takes place.

## Methods

### Fly Strains, Media, and Larval Growth

The fly rearing and the egg collections were performed as previously described^32^. After a 2.5-h synchronized egg collection, eggs were kept in a 12 hour light/dark cycle in an incubator at 25 °C. Larvae were transferred to a fresh apple juice plate with yeast paste at the age of 48–52 h (<80 larvae per plate). The fly lines used included *Oamb* ^286^ ^33,34^, *Oamb* ^*MB00297*^ (BL22758)^35,36^, UAS*-GFP*.*nls* (BL4775), UAS*-mCD8-GFP* (BL32184), *GS-elav-GAL4* (BL43642)^37^, UAS*-Octβ2R* ^*dsRNA*^ (BL34673), UAS*-Octβ3R* ^*dsRNA*^ (BL31108), *TH-GAL4* ^38^, *VGlut-GAL4* (BL24635), *TRH-GAL4* (BL38388), *dIlp2-GAL4* (BL37516). UAS*-Oamb* ^*dsRNA*^ (#2861)^39^, UAS*-oa2* ^*dsRNA*^ (#47896)^39^ were obtained from the Vienna Drosophila RNAi Center.

### Transgenic Constructs

A 1.8 kb genomic DNA fragment containing the 5′ regulatory region of *Octβ3R* was cloned by PCR with two the primers, 5′-AGGTGACACACACCACATCG-3′ and 5′-CTGAGTCTCGGCCAAGTCC-3′. The Octβ3R-GAL4 construct was made by subcloning the PCR product into the pCaSpeR-GAL4 vector at the EcoR I site.

To construct the Oamb-GAL4 driver line, a 1.6 kb DNA fragment containing the 5′ regulatory sequence for the Oamb gene was amplified by 5′-ATACATACTAGAATTCTCTGAAAGCTGCGGGATA-3′ and 5′-GGGCGAGCTCGAATTCCGGCAAGAACCGTTAGTTC-3′ and cloned into the pCaSpeR-GAL4 vector at the EcoR I site. The purified construct was injected to w1118 background (BestGene Inc).

### Behavioral Assays

All assays were quantified under blind conditions. The rate of larval food intake was quantified by following a previously published protocol with slight modifications^6,40^. 10% (W/W) glucose food was prepared by mixing 45 ml ddH_2_O, 5 g D-glucose (Fisher Chemical), and 6 g agar powder (US Biological). 3% (V/V) fatty acid food was prepared by mixing 45 ml ddH_2_O, 1.4 ml oleic acid (Sigma-Aldrich), and 6 g agar powder. 0.5% (W/W) tryptone food was prepared by mixing 45 ml ddH_2_O, 0.23 g tryptone (Sigma-Aldrich), and 6 g agar powder. For assays, 10 to 20 early third-instar larvae were transferred to the center of the assay plate, and then each plate was videotaped for 2 min. The number of MHCs per 30 s was scored and analyzed.

The feeding assay was performed in a 35-mm Petri dish containing 0.5 g of food paste. The food ingestion assay was performed by feeding a group of 30 larvae 10% (W/W) glucose liquid media prepared as above containing 1% food dye FD&C No. 1 (Sigma-Aldrich) for 3 minutes. Larvae were removed from the food and rinsed with a copious amount of water, then were quickly frozen in liquid nitrogen and homogenized in 100 uL 0.1 M phosphate buffer (pH 7.2). The homogenates were centrifuged at 30,000 × g for 10 minutes and supernatants were analyzed with a spectrophotometer for absorbance at 625 nm. At least three separate trials were used for each line, with untreated larvae run in simultaneous batches to provide control for background absorbance measures. The data presented are normalized to background signal of un-dyed larvae.

### Immunohistochemistry

Brains from larvae 76 h after egg lay were dissected out and the immunostaining were performed as previously described^12^ by using chicken anti-GFP (1:1,000; Invitrogen), Alexa 488-goat anti-chicken (1:2,000; Invitrogen). Images were collected using a Zeiss LSM510 META confocal microscope.

### Statistic analyses

Statistical analyses for feeding and ingestion assays were performed using Kruskal-Wallis test followed by Dunn’s multiple comparisons test or one-way ANOVA followed by Tukey’s or Dunnett’s multiple comparisons test.

## Electronic supplementary material

Supplementary Information
