# Supplementary material for: Genetic and Neurobiological Analyses of the Noradrenergic-like System in Vulnerability to Sugar Overconsumption Using a Drosophila Model
Source: Sci Rep. 2017 Dec 15;7:17642. doi: 10.1038/s41598-017-17760-w (PMC5732301; doi:10.1038/s41598-017-17760-w)
Supplement: Supplementary file 1 — Supplementary Information [file 41598_2017_17760_MOESM1_ESM.pdf]

Genetic and Neurobiological Analyses of the Noradrenergic-like System in Vulnerability to Sugar Overconsumption Using a *Drosophila* Model

Audrey Branch[1], Yiwen Zhang[1], and Ping Shen[†]

Department of Cellular Biology and Biomedical and Health Sciences Institute, University of Georgia, 500 D. W. Brooks Drive, Athens, GA 30602, USA.

1 co-first authors

† Correspondence should be addressed to P.S. (pshen@uga.edu).

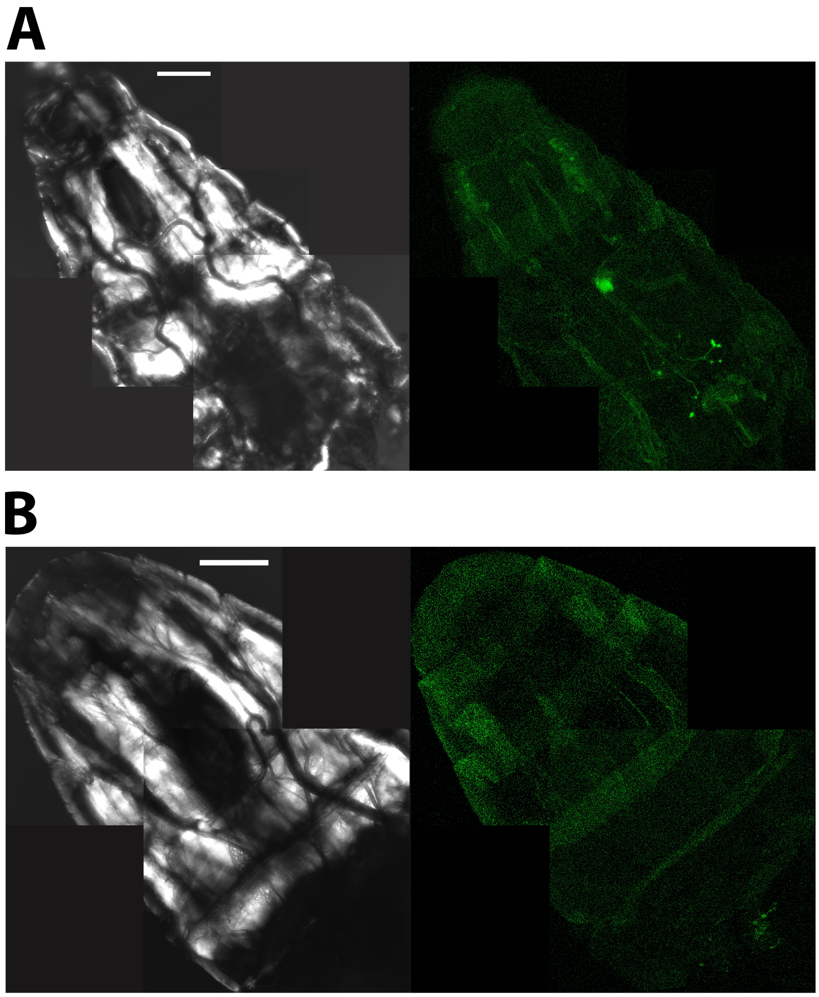

Supplementary Figure S1 Analysis of GFP expression in larval peripheral nervous systems under the direction of 1.6-*Oamb-GAL4* or 1.8-*Octβ3R-GAL4.* (**A**) A filleted larval tissue expressing a GFP reporter driven by 1.6-*Oamb-GAL4.* (**B**) A filleted larval tissue expressing a GFP reporter driven by 1.8-*Octβ3R-GAL4*. Both larvae showed no detectable GFP signals in the peripheral neurons including gustatory and olfactory neurons and nerves that innervate the larval brain and ventral ganglia. (**C**) As a control, it shows that the intact CNS tissue from the same larvae used in the first panel expresses a GFP reporter driven by 1.6-*Oamb-GAL4.* Scale bar=150um.
